# Intracrine Androgens Enhance Decidualization and Modulate Expression of Human Endometrial Receptivity Genes

**DOI:** 10.1038/srep19970

**Published:** 2016-01-28

**Authors:** Douglas A. Gibson, Ioannis Simitsidellis, Fiona L. Cousins, Hilary O. D. Critchley, Philippa T. K. Saunders

**Affiliations:** 1 Medical Research Council Centre for Inflammation Research, The University of Edinburgh, Queen’s Medical Research Institute, 47 Little France Crescent, Edinburgh, EH16 4TJ. UK; 2 Medical Research Council Centre for Reproductive Health, The University of Edinburgh, Queen’s Medical Research Institute, 47 Little France Crescent, Edinburgh, EH16 4TJ. UK

## Abstract

The endometrium is a complex, steroid-dependent tissue that undergoes dynamic cyclical remodelling. Transformation of stromal fibroblasts (ESC) into specialised secretory cells (decidualization) is fundamental to the establishment of a receptive endometrial microenvironment which can support and maintain pregnancy. Androgen receptors (AR) are present in ESC; in other tissues local metabolism of ovarian and adrenal-derived androgens regulate AR-dependent gene expression. We hypothesised that altered expression/activity of androgen biosynthetic enzymes would regulate tissue availability of bioactive androgens and the process of decidualization. Primary human ESC were treated *in vitro* for 1–8 days with progesterone and cAMP (decidualized) in the presence or absence of the AR antagonist flutamide. Time and treatment-dependent changes in genes essential for a) intra-tissue biosynthesis of androgens (5α-reductase/SRD5A1, aldo-keto reductase family 1 member C3/AKR1C3), b) establishment of endometrial decidualization (IGFBP1, prolactin) and c) endometrial receptivity (SPP1, MAOA, EDNRB) were measured. Decidualization of ESC resulted in significant time-dependent changes in expression of AKR1C3 and SRD5A1 and secretion of T/DHT. Addition of flutamide significantly reduced secretion of IGFBP1 and prolactin and altered the expression of endometrial receptivity markers. Intracrine biosynthesis of endometrial androgens during decidualization may play a key role in endometrial receptivity and offer a novel target for fertility treatment.

In fertile women the endometrium ‘prepares’ for implantation of a blastocyst by a period of post-ovulatory (progesterone-dependent) tissue remodelling. This process is controlled by endometrial stromal cells (ESC) that undergo decidualization, a process of differentiation, that results in transformation from proliferating fibroblasts to specialised secretory cells capable of producing factors that promote endometrial receptivity and regulate multiple cell types including those of the immune and vascular systems^1^. Recent studies from our laboratory have revealed that decidualization of ESC results in significant changes in biosynthesis and metabolism of estrogens (estrone and estradiol)^2^ which alters the function of uterine natural killer and endometrial endothelial cells^3^. These results have prompted us to propose a pivotal role for intra-uterine steroid biosynthesis in the establishment of a receptive endometrium. Androgens are natural precursors to estrogens but can also regulate functional processes through binding and activating the androgen receptor (AR). In the current study we have investigated whether tissue specific synthesis of androgens can play a role in regulation of decidualization.

The human endometrium is an androgen target tissue and androgen receptors (AR) are expressed in the endometrium throughout the menstrual cycle. Our detailed analysis of full thickness sections of human endometrium has revealed intense regional and stage-dependent immunoexpression in ESC^4^ highlighting them as a key target for androgen action. In women, androgens are secreted both by the ovary and the adrenal gland^5^ with production declining with age^6^. Circulating concentrations of androgens are high throughout the menstrual cycle with a mid-cycle peak in concentrations of the AR agonist testosterone (T) at the time of ovulation^7^. Circulating concentrations of dihydrotestosterone (DHT) are low and do not change across the cycle^7^ but this is not unexpected as DHT is primarily a product of peripheral T metabolism within target tissues and thus there is a poor correlation between circulating and tissue-specific concentrations of DHT. The adrenal androgen dehydroepiandrosterone (DHEA) and its sulphate (DHEAS) are abundant in the circulation and can act as a precursor to both estrogens and androgens (Supplementary Figure 1). DHEA is converted to androstenedione (A4) by the action of the enzyme 3-β-hydroxysteroid dehydrogenase (3βHSD) which we have previously reported is expressed in decidualized endometrium and in isolated ESC^2^. A4 is a weak AR agonist but can be converted to T by the aldo-keto reductase family 1 member C3 (AKR1C3; also known as 17β-HSD5). T can act as a precursor for estrogen biosynthesis by the action of aromatase (encoded by *CYP19A1*)^2^ or converted to the potent non-aromatisable androgen DHT via the action of 5α-reductase type 1 (encoded by *SRD5A1*). In the 1970s Rose *et al*. detected conversion to DHT in human endometrial homogenates co-incubated with radiolabelled testosterone^8^. Recent evidence using advanced liquid chromatography-tandem mass spectrometry (LC-MS/MS) has extended these initial observations by demonstrating that intra-tissue hormone concentrations in endometrial homogenates are distinct from circulating concentrations^9^,^10^ consistent with a potential role for local activation of androgens within the endometrium.

Primary stromal cells isolated from human endometrium (ESC) can be stimulated to undergo a robust decidualization response by the addition of progesterone and the second messenger molecule cyclic adenosine monophosphate (cAMP)^11^. This *in vitro* decidualization phenocopies the post-ovulatory differentiation of ESC during a fertile cycle with characteristic changes in cellular morphology^11^ and increased secretion of proteins such as insulin-like growth factor-binding protein 1 (IGFBP1)^12^ and prolactin^13^. Some *in vitro* studies have used the synthetic progestin medroxyprogesterone acetate (MPA) in place of progesterone. As MPA is reported to activate the AR, in addition to its well-known role as a progesterone receptor (PR) agonist^14^, changes in gene expression detected using this agent may represent a combination of PR- and AR-dependent effects. Additional evidence from studies comparing the behaviour of human ESC decidualized with progesterone plus cAMP combined with the addition of exogenous DHT have reported that regulation of prolactin secretion, morphological transformation of ESC and resistance to oxidative stress are augmented by androgen action^15^,^16^,^17^. Genomic studies using *in silico* and knockdown approaches have reported that DHT and AR-dependent signaling can regulate distinct gene networks in decidualized human ESC with evidence for a role in cell survival, cell cycle regulation and cytoskeletal organisation^4^,^18^. Studies conducted in rodents suggest androgens play an important role in the establishment and maintenance of pregnancy in those species. In rats administration of the AR antagonist flutamide is reported to suppress decidualization and delay initiation of implantation^19^. Female mice lacking a functional *Ar* have altered neuroendocrine signalling and ovarian function and are subfertile^20^; studies using ovarian transplantation suggest *Ar* plays a key role in uterine development^21^. Taken together, these data are consistent with the uterus being an androgen target tissue and that AR-dependent signalling is essential for the regulation of uterine function.

In androgen target tissues local activation of circulating precursors regulate AR-dependent gene expression but whether local metabolism and activation of androgens occurs within the endometrium is not known. In the current study we tested the hypothesis that changes in intra-uterine bioavailability of androgens would have a direct impact on endometrial tissue function. We demonstrate that decidualization of ESC is associated with local biosynthesis of androgens. Our novel data demonstrate that intracrine androgens can complement the effects of progesterone by acting to enhance the decidualization response and support an optimal environment for successful establishment of pregnancy.

## Results

### Decidualization is associated with biosynthesis of testosterone

**Figure 1:**
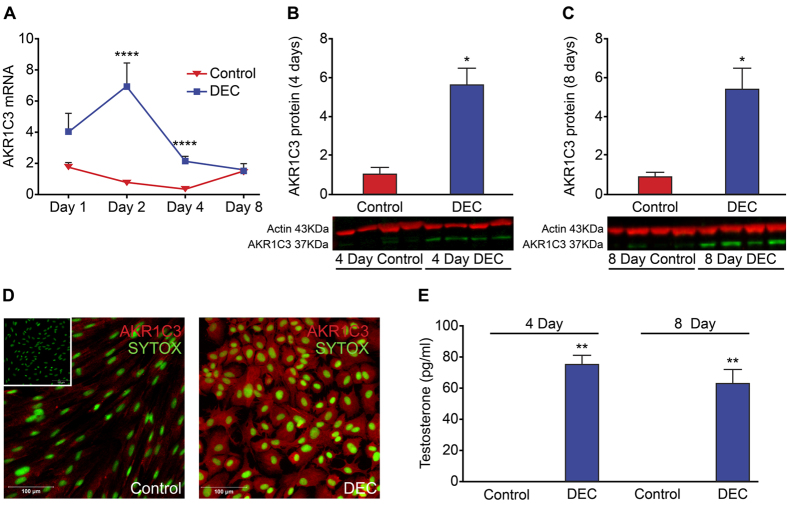
Decidualization is associated with time-dependent changes in expression of *AKR1C3* and biosynthesis of testosterone. The expression of the androgen biosynthetic enzyme AKR1C3, which converts A4 to T, was assessed by qPCR, Western blot and immunocytochemistry in ESC. AKR1C3 was increased following decidualization of ESC. (**A**) Concentrations of mRNAs encoding *AKR1C3* were significantly increased in ESC treated to decidualized (DEC) for 2 and 4 days compared to control (Control, p < 0.0001, n = 8) but by day 8 mRNA expression was unchanged between control and decidualized ESC. (**B**) Western blot analysis of AKR1C3 expression from homogenates of ESC treated for 4 days revealed a significant increase in AKR1C3 protein in decidualized ESC (n = 4 patients per treatment, p < 0.05). (**C**) Western blot analysis of AKR1C3 expression from homogenates of ESC treated for 8 days revealed a significant increase in AKR1C3 protein in decidualized ESC (n = 6 patients per treatment, p < 0.05). Representative blots from 4 matched patients are shown (**B**,**C**). Loading control b-actin (red, 43 kDa), AKR1C3 (green, 37 kDa). (**D**) The expression of AKR1C3 was assessed by immunocytochemistry in ESC grown in chamber slides and treated to decidualize for 4 days. AKR1C3 expression was detected in both control and decidualized ESC (AKR1C3; red staining). Staining appeared more intense in decidualized ESC (DEC). Nuclear counterstain SytoxGreen (SYTOX; green staining), scale bar 100 μm. In the absence of primary antibody no staining was detected (inset). (**E**) Concentrations of T were assessed by ELISA and significant concentrations of T were detected in cell culture supernatants recovered from decidualized ESC at 4 (n = 8, p < 0.01) and 8 days (n = 8, p < 0.01). *p < 0.05, **p < 0.01, ****p < 0.0001.

The expression of AKR1C3 was dynamically regulated across the decidualization time course. Concentrations of *AKR1C3* mRNA were increased in ESC treated with decidualization media (DM) for 1, 2 and 4 days with a significant peak in expression detected after 2 and 4 days of treatment (Fig. 1A, n = 8 p < 0.0001). Western blot analysis revealed a significant increase in total AKR1C3 protein in decidualized ESC compared to control after 4 (Fig. 1B, n = 8 p < 0.05) and 8 days (Fig. 1C, n = 8 p < 0.05). Immunofluorescence revealed positive staining for AKR1C3 (red) immunolocalised in the cytoplasm of both control and decidualized ESC treated for 4 days (Fig. 1D) although staining was more intense in decidualized cells consistent with the pattern of expression detected by Western blot. A significant increase in concentrations of T was detected in media recovered after 4 and 8 days of decidualization compared with controls (Fig. 1E, n = 8 p < 0.01)). Metabolism of testosterone to A4 was assessed by TLC and was found to be significantly decreased in decidualized cells further augmenting net production of T (Supplementary Figure 2; p < 0.05).

### Decidualization is associated with time-dependent changes in expression of SRD5A1 and biosynthesis of dihydrotestosterone

**Figure 2:**
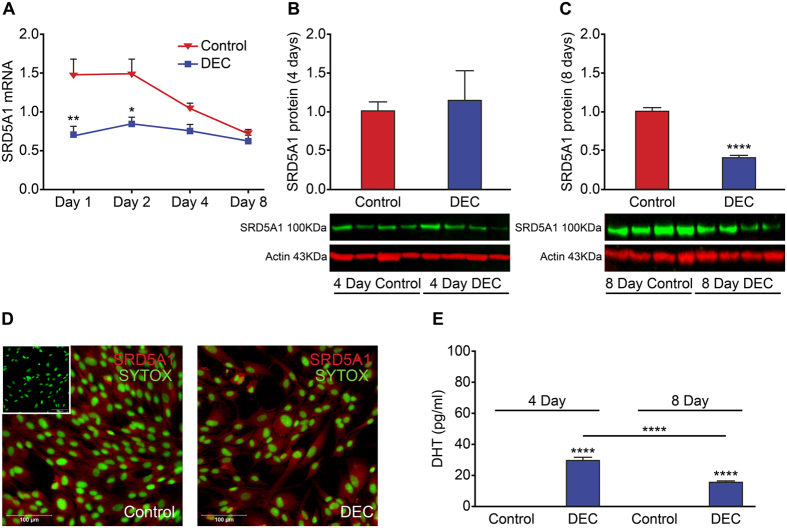
Decidualization is associated with time-dependent changes in expression of *SRD5A1* and biosynthesis of dihydrotestosterone. The expression of the androgen biosynthetic enzyme SRD5A1, which reduces T to the more potent androgen DHT, was assessed by qPCR, Western blot and immunocytochemistry in ESC. (**A**) Concentrations of mRNAs encoding *SRD5A1* were significantly decreased in ESC treated with DM for 1 and 2 days compared to control (Control, p < 0.01 and p < 0.05 respectively, n = 8) but by day 8 mRNA expression was unchanged between control and decidualized ESC. (**B**) Western blot analysis of 5α-reductase (SRD5A1) expression from homogenates of ESC treated for 4 days revealed that 5α-reductase protein in was detected in control and decidualized ESC (n = 4 patients per treatment) (**C**) Western blot analysis of 5α-reductase expression from homogenates of ESC treated for 8 days revealed a significant decrease in 5α-reductase protein in decidualized ESC (n = 7 patients per treatment, p < 0.0001), representative blots from 4 matched patients are shown (**B**,**C**). Loading control b-actin (red, 43 kDa), 5α-reductase (green, 100 kDa). (**D)** The expression of 5α-reductase was assessed by immunocytochemistry in ESC grown in chamber slides and treated to decidualize for 4 days. 5α-reductase expression was detected in both control and decidualized ESC (SRD5A1; Red staining). Staining appeared to be similar in both control and decidualized ESC (DEC). Nuclear counterstain SytoxGreen (SYTOX; green staining), scale bar 100 μm. In the absence of primary antibody no staining was detected (Inset). (**E**) Concentrations of DHT were assessed by ELISA and significant concentrations of DHT were detected in cell culture supernatants recovered from decidualized ESC at 4 (n = 8, p < 0.0001) and 8 days (n = 8, p < 0.0001). Concentrations of DHT were significantly decreased in day 8 compared to day 4 decidualized ESC (p < 0.001). *p < 0.05, **p < 0.01, ****p < 0.0001.

The enzyme SRD5A1 catalyses the conversion of T to the potent endogenous androgen DHT. Interestingly, the expression of *SRD5A1* mRNAs were significantly decreased after 1 (Fig. 2A; n = 8 p < 0.01) and 2 days (Fig. 2A; n = 8 p < 0.05) but unchanged compared to control at 8 days. Western blot analysis revealed expression of SRD5A1 in cell lysates from both control and decidualized cells that was unchanged after 4 days of treatment (Fig. 2B) but significantly decreased in decidualized ESC compared to control after 8 days of treatment (Fig. 2C, n = 7 p < 0.0001). Immunofluorescence staining of ESC treated for 4 days confirmed positive staining for SRD5A1 (red) immunolocalised in the cytoplasm of both control and decidualized ESC (Fig. 2D). Although SRD5A1 protein was detected in both control and decidualized ESC, significant concentrations of DHT were only detected in supernatants from decidualized ESC (Fig. 2E, n = 8 p < 0.0001); concentrations of DHT were significantly decreased in day 8 compared to day 4 decidualized ESC (Fig. 2E, n = 8 p < 0.001).

### Blocking intracrine androgen action with flutamide inhibits decidualization

**Figure 3:**
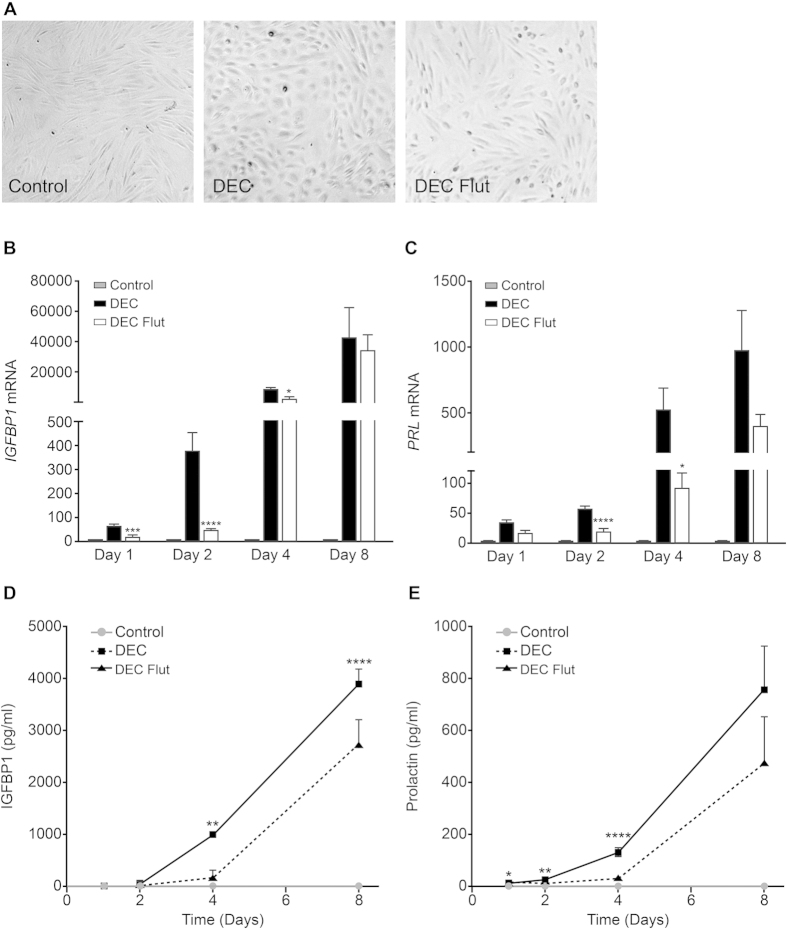
Blocking local androgen action inhibits decidualization. To investigate if androgens produced by ESC could affect decidualization, ESC were co-treated with the anti-androgen flutamide and markers of decidual transformation were assessed. (**A**) Cell morphology was assessed in ESC by phase-contrast microscopy. Control ESC had an elongated fibroblast-like morphology and decidualized (DEC) ESC had a rounded, ‘epithelioid’ morphology. Flutamide treated cells (DEC Flut) were mostly fibroblast-like with few rounded cells. (**B**) Concentrations of mRNAs encoding *IGFBP1* were significantly reduced after 1 (n = 6, p < 0.001), 2 (n = 6, p < 0.0001) and 4 days (n = 6, p < 0.05) of flutamide treatment but were unchanged after 8 days. (**C**) Concentrations of mRNAs encoding PRL were significantly decreased after 2 (n = 6, p < 0.0001) and 4 days (n = 6, p < 0.05) of flutamide treatment and tended to be lower in flutamide treated ESC after 8 days. (**D**) Secretion of the decidualization marker IGFBP1 was assessed by ELISA. Secretion of IGFBP1 was significantly reduced by flutamide treatment (DEC Flut). A striking 80% reduction in secretion of IGFBP1 was detected at 4 days (n = 6, p < 0.01) and 30% reduction was detected after 8 days of flutamide treatment (n = 6, p < 0.0001) compared to DEC. (**E**) Secretion of Prolactin was significantly reduced by flutamide treatment after 1 (n = 6, p < 0.05) and 2 days (n = 6, p < 0.01). A striking 81% reduction in secretion of Prolactin was detected at 4 days (n = 6, p < 0.0001) and secretion of Prolactin tended to be lower in flutamide treated ESC after 8 days compared to DEC ESC (n = 6). *p < 0.05, **p < 0.01, ***p < 0.001, ****p < 0.0001.

As decidualization of ESC resulted in biosynthesis of T and DHT we investigated whether these androgens could act as intracrine regulators of decidualization. Markers of decidual transformation were compared in ESC incubated with control media, DM or DM plus the specific AR antagonist flutamide. Cell morphology was assessed in ESC and control cells had an elongated fibroblast-like morphology and decidualized (DEC) ESC exhibited classic rounded, ‘epithelioid’ morphology (Fig. 3A). Consistent with inhibition of decidualization, flutamide treated cells (DEC Flut) exhibited limited morphological evidence of decidualization with mostly fibroblast-like morphology and few rounded cells (Fig. 3A). The presence of flutamide had a significant impact on expression of both IGFBP1 and prolactin: Concentrations of mRNAs encoded by *IGFBP1* were significantly reduced by flutamide treatment after 1 (Fig. 3B; n = 6 p < 0.001), 2 (p < 0.0001) and 4 days (p < 0.05). *PRL* mRNA was significantly decreased at 2 (Fig. 3C; n = 6 p < 0.0001) and 4 days (Fig. 3C; n = 6 p < 0.05). Mean concentrations of secreted IGFBP1 protein detected in culture media from cells incubated with flutamide were significantly decreased after 4 days (Fig. 3D; n = 6 p < 0.01) and 8 days (Fig. 3D; n = 6 p < 0.0001) compared to DEC alone. Mean concentrations of secreted Prolactin protein detected in culture media from cells incubated with DM plus flutamide were significantly decreased after 1, 2 and 4 days (Fig. 3E; n = 6 p < 0.05, p < 0.01, p < 0.0001) compared to DM alone. Notably, mean concentrations of secreted IGFBP1 and Prolactin were decreased approximately 80% by flutamide treatment after 4 days.

### Addition of flutamide altered expression of putative androgen-regulated receptivity genes

**Figure 4:**
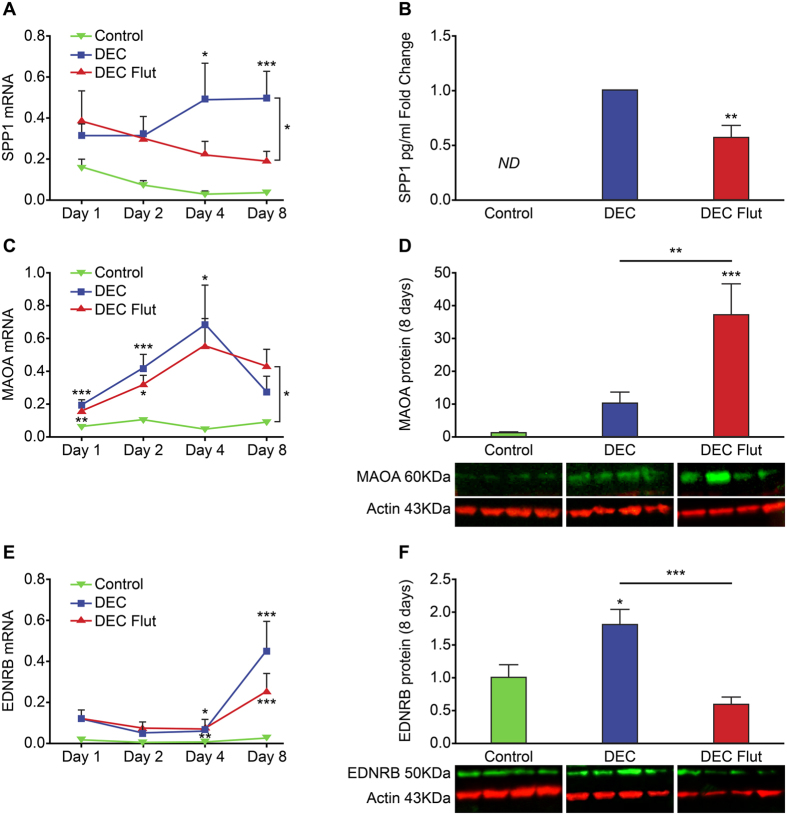
The impact of flutamide on expression of putative androgen-regulated receptivity genes. To investigate if androgens produced by ESC could affect expression of putative androgen-regulated endometrial receptivity genes, ESC were co-treated with the anti-androgen flutamide and the expression of osteopontin (SPP1), monoamine oxidase (MAOA) and endothelin receptor B (EDNRB) were assessed. (**A**) Concentrations of mRNAs encoding *SPP1* were significantly increased in decidualized ESC after 4 (p < 0.05) and 8 days treatment (p < 0.001) and significantly reduced in ESC co-treated with flutamide (n = 8, p < 0.05) at 8 days. (**B**) Concentrations of secreted SPP1 were detected by ELISA and calculated as fold change relative to decidualized ESC. SPP1 was not detected in supernatants from control cultures (ND). Co-treatment with flutamide significantly reduced secretion of SPP1 (n = 8, p < 0.01) and reduced the relative secretion of SPP1 by ~40%. (**C**) Concentrations of mRNAs encoding *MAOA* were significantly increased in decidualized ESC after 1 (p < 0.001), 2 (p < 0.001) and 4 days treatment (p < 0.05). *MAOA* mRNA expression was significantly increased relative to control in ESC co-treated with flutamide for 8 days (n = 8, p < 0.05). (**D**) Western blot analysis of MAOA expression from homogenates of ESC treated for 8 days revealed that MAOA protein was detected in control and decidualized ESC (n = 8 patients per treatment), and significantly increased in ESC co-treated with flutamide relative to control and decidualized ESC (n = 8, p < 0.001 and p < 0.01). Representative blots from 4 matched patients are shown. Loading control b-actin (red, 43 kDa), MAOA (green, 60 kDa). (**E**) Concentrations of mRNAs encoding *EDNRB* tended to be increased at each time point and were significantly increased in decidualized ESC after 8 days treatment (n = 8, p < 0.001). *EDNRB* mRNA expression was reduced in ESC co-treated with flutamide (**F**) Western blot analysis of EDNRB expression from homogenates of ESC treated for 8 days revealed that EDNRB protein was detected in control and decidualized ESC (n = 8 patients per treatment), and significantly increased in ESC treated to decidualize (p < 0.05). Co-treatment with flutamide significantly reduced concentrations EDNRB detected relative to decidualized ESC (n = 8, p < 0.001). Representative blots from 4 matched patients are shown. Loading control b-actin (red, 43 kDa), EDNRB (green, 50 kDa). *p < 0.05, **p < 0.01, ***p < 0.001, ****p < 0.0001.

Candidate receptivity genes were selected by comparing androgen-regulated genes^4^ with genes previously validated as playing an essential role in endometrial receptivity^22^,^23^. Incubation of ESC with DM resulted in significant time-dependent changes in concentrations of mRNAs and proteins encoded by *SPP1*, *MAOA* and *EDNRB*: changes induced by addition of flutamide were consistent with a role for androgens in regulation of these 3 genes. Osteopontin/Secreted Phosphoprotein 1 (SPP1) is reported to be an essential mediator of implantation and receptivity in women^24^,^25^ and is reported to be increased in decidualized ESC^26^. *SPP1* mRNA was significantly increased in ESC treated to decidualize (DEC) for 4 (Fig. 4A; n = 8 p < 0.05) and 8 days (Fig. 4A; n = 8 p < 0.001). Concentrations of *SPP1* mRNA were lower in cells treated with flutamide and this was significant at the 8-day time point (Fig. 4A; n = 8 p < 0.05). SPP1 is a phosphoprotein which is secreted in active form from cells to mediate effects in tissues. Concentrations of SPP1 protein in cell culture supernatants was therefore assessed by ELISA. Interestingly, the concentrations of SPP1 detected in culture media varied widely between patients but were generally lower after 8 days flutamide treatment compared to DEC (Supplementary Figure 3) and undetectable in control supernatants. To account for inter-patient variability, protein concentrations were calculated as fold change relative to DEC which revealed SPP1 secretion was significantly decreased with flutamide treatment at 8 days (Fig. 4B, n = 8 p < 0.01).

Incubation with DM alone significantly increased monoamine oxidase (*MAOA)* mRNA concentrations after 1 (Fig. 4C; n = 8 p < 0.001), 2 (Fig. 4C; n = 8 p < 0.001) and 4 days (Fig. 4C; n = 8 p < 0.05) but was not significantly different from controls at 8 days. Interestingly, the impact of flutamide treatment on *MAOA* mRNA expression was also time-dependent; on days 1–4 addition of flutamide had no effect on *MAOA* mRNAs compared to DM alone but on day 8 concentrations were significantly increased in ESC treated with flutamide compared to control (Fig. 4D; n = 8, p < 0.05). Western blot analysis of MAOA revealed that addition of flutamide had a striking effect on total protein concentrations in cells on day 8 compared to both control (Fig. 4D, n = 8, p < 0.001) and DM alone (Fig. 4D, n = 8, p < 0.01).

Endothelin receptor B (EDNRB) binds members of the endothelin family of proteins that are reported to regulate endometrial blood flow^27^. The expression of EDNRB was also dynamically regulated during decidualization of ESC. Decidualization led to a significant increase in the concentrations of *EDNRB* mRNAs in ESC treated to decidualize for 4 (Fig. 4E, n = 8 p < 0.05) and 8 days (Fig. 4E, n = 8 p < 0.001). Incubation with flutamide resulted in an apparent (non-significant) decrease in *EDNRB* mRNA compared to DM alone although concentrations were increased relative to control at 4 (Fig. 4E, n = 8 p < 0.01) and 8 days (Fig. 4E, n = 8 p < 0.001). On Western blots EDNRB protein was readily detected in all treatment groups, was significantly increased after 8 days treatment with DM (Fig. 4F, n = 8 p < 0.05) but this increase was completely abrogated by inclusion of flutamide (Fig. 4F, n = 8 p < 0.001; DEC vs DEC Flut). Flutamide treatment also decreased EDNRB protein expression after 4 days of treatment compared to DM alone (Supplementary Figure 4A, n = 4 p < 0.05) however MAOA was not detected by Western blot at this time point (Supplementary Figure 4B).

## Discussion

**Figure 5:**
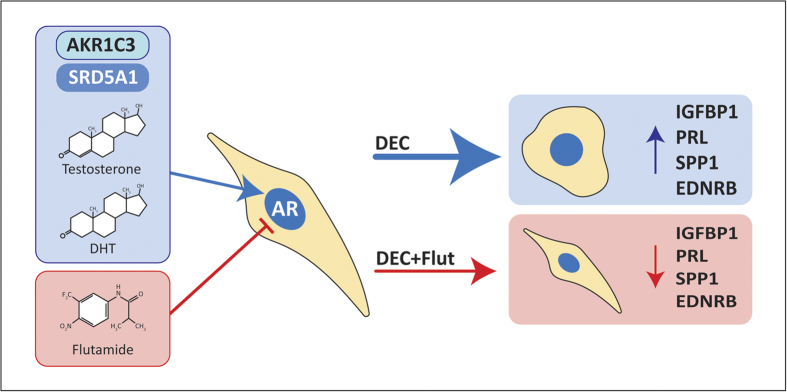
Summary: The impact of intracrine androgens on endometrial function. Decidualization of stromal cells is a time-dependent process that is associated with changes in synthesis of bioactive steroids. During the first 4 days of decidualization increased expression and activity of AKR1C3 promotes biosynthesis of T which is converted to the potent androgen DHT by the action of SRD5A1. T and DHT produced by ESC signal via an intracrine mechanism to regulate the early decidualization response leading to characteristic transformation of cellular morphology and secretion of decidualization markers. Blocking intracrine androgens with the AR antagonist flutamide inhibits decidualization, limiting the morphological transformation of ESC and inhibiting the production of decidualization (IGFBP1 and prolactin) and receptivity factors (SPP1, EDNRB). Inadequate T/DHT biosynthesis may result in a transcriptional profile that is ‘out of phase’ which may impact on the establishment and maintenance of pregnancy in women.

Endometrial receptivity is a time-sensitive process underpinned by dynamic changes in the transformation of the stromal compartment and coordinated stromal-epithelial cross-talk. To our knowledge we present the first data that demonstrate endometrial stromal cells promote formation of a pro-androgenic microenvironment during decidualization. Critically, we found that changes in androgen metabolism and biosynthesis are time-dependent, suggesting androgens will be most abundant within areas of the tissue at early stages of decidualization. We found that incubation with the selective AR antagonist flutamide had a significant effect on gene expression within primary human ESC over the time course of decidualization. Notably in this study we did not add exogenous T or DHT therefore the impact of flutamide on gene transcription and protein biosynthesis provides evidence of intracrine androgen activity. We noted that blocking intracrine androgens both inhibited and delayed expression of proteins implicated as markers of the decidualization response and endometrial receptivity (Summarised in Fig. 5).

The human endometrium has been extensively investigated using transcriptional profiling^22^,^23^. A customised array based on 238 genes (The Endometrial Receptivity Array (ERA)^22^) has been validated and is in current use in the clinic to optimise management and timing of embryo transfer for women undergoing assisted reproduction, including those with repeated implantation failure. In studies using primary ESC our group has previously identified and validated a set of androgen responsive genes that were expressed in the human endometrium during the normal cycle^4^. In the present study we identified putative androgen-regulated receptivity genes by cross-referencing the ERA gene list^22^ with our ‘androgen target gene’ set^4^. Three putative androgen-regulated genes were identified; *MAOA*, *EDNRB* and *SPP1*. Blocking local androgen action resulted in time-dependent inhibition of SPP1 and EDNRB expression and altered expression of MAOA in decidualized ESC. Decidualization of ESC precedes implantation in the human endometrium and thus the expression of receptivity factors such as SPP1, MAOA and EDNRB may be affected by inadequate decidualization and alterations in androgen signalling. Expression of MAOA is reported to be enhanced in the endometrium during the window of implantation and deficient expression is associated with implantation failure^28^. SPP1 is the only factor identified that is common to all reported endometrial receptivity gene sets^22^,^23^ and is reported to be an essential mediator of implantation and receptivity in humans^24^,^25^. The novel findings in the present study demonstrate that biosynthesis of androgens within endometrial tissue during the early secretory phase may play an essential role in maintaining SPP1 and EDNRB expression in ESC which could have important implications for the regulation of implantation. Timing of implantation and endometrial receptivity has a major impact on the establishment of a successful pregnancy and on the future health of offspring. It has been reported that the majority of conceptions can be detected between days 22 and 24 of the menstrual cycle^29^ and that delayed implantation is associated with increased rates of early pregnancy loss^30^,^31^. The data in the current study suggest that a deficit in local bioavailable androgens may delay endometrial maturation and might be a previously unrecognised factor contributing to a transcriptional profile that is ‘out of phase’.

Insufficient androgen signalling may be detrimental to the regulation of decidualization however exposure to excess androgens may also adversely affect endometrial function. Elevated AR expression has been reported in endometrium of women with polycystic ovarian syndrome (PCOS)^32^,^33^ a complex metabolic disorder associated with ovarian dysfunction, irregular menstrual cycles and in some cases hyperandrogenism. In a recent study, Piltonen *et al*. reported that in ESC isolated from women with PCOS (fulfilling all Rotterdam criteria; oligo-amenorrhea, PCO and hyperandrogenism) response to decidualization induced by estradiol (E2) and progesterone was significantly reduced, however this was only in a small subset of the patients that were analysed^32^. These results suggest that altered androgen signalling may have a detrimental effect on endometrial function in women, however further studies are needed to elucidate the importance of these effects in complex disorders such as PCOS. Notably, complementary studies in rodents demonstrate that insufficient or excess concentrations of androgens can delay implantation or lead to aberrant gene expression in implantation sites respectively^33^.

The widely reported age-related decline in fertility is often considered as an exclusively ovarian phenomenon but there is emerging evidence that reduced androgen bioavailability may result in endometrial ‘senescence’ a phenomenon that has received little attention to date. Concentrations of circulating androgens decline precipitously with age^34^, such that concentrations of adrenal androgens at age 40 are half those at age 21^6^. Changes in the availability of precursor androgens in circulation may impact on intracrine signalling in the endometrium and thus the regulation of implantation. We have previously reported that ESC express 3βHSD the enzyme responsible for converting adrenal androgens within target tissues^2^ and we speculate that a reduction in the availability of circulating androgen precursors could limit the development of a full and robust decidualization response which may contribute to reduced fertility in older women. Studies evaluating the effect of DHEA supplementation on reproductive outcomes have produced conflicting results and have been limited by a lack of randomised controlled trials (RCT). A recent meta-analysis pooling data from 8 studies (including two RCTs) investigated the impact of DHEA supplementation on clinical pregnancy rate and number of oocytes retrieved in women with diminished ovarian reserve undergoing *in vitro* fertilisation or intracytoplasmic sperm injection^35^. The study reported that DHEA supplementation significantly increased clinical pregnancy rate but there was no significant effect on oocyte retrieval, implantation or abortion^35^. These findings are in agreement with the conclusions of the recent Cochrane review on androgen/DHEA supplementation for women undergoing assisted reproduction that reported that DHEA or testosterone supplementation may be associated with improved live birth rates but further evidence from well-designed studies is required before definitive conclusions can be made^36^.

In the current study we provide further novel insights into the regulation of decidualization by profiling discrete responses of ESC across a time-course up to 8 days whereas many other studies only consider the response of cells after 6 or more days incubation^15^,^26^. We demonstrate rapid and sustained induction of AKR1C3 resulting in a significant increase in secretion of T which was maintained throughout the culture period. Notably, concentrations of DHT in media were significantly higher on day 4 than day 8 which may in part reflect reduced expression of SRD5A1. An alternative explanation for the reduced concentrations of DHT would be a switch towards conversion of T to E2 a hypothesis that would be consistent with our recent study in which we reported that E2 biosynthesis increases in a time-dependent manner during decidualization due to an increase in expression of aromatase *(CYP19A1*) by 8 days decidualization^2^. Furthermore, we have demonstrated that local E2 plays an important role in the endometrium by regulating immune and vascular function in early pregnancy^3^. These complementary data suggest perturbations in formation of androgens and the androgen-estrogen balance could have a major impact on the endometrial microenvironment as a result of intracrine androgen regulation of stromal decidualization and paracrine estrogen regulation of immune-vascular function. Taken together these data indicate that T may play a dual role in the endometrium as both a precursor to DHT in early decidualization and as a precursor to estrogens as decidualization progresses and pregnancy is established.

The results of this study suggest a new paradigm for understanding control of endometrial remodelling in which local androgens modulate decidualization and promote coordinated development of the endometrial microenvironment. Critically, we describe a novel role for androgens that is both distinct from and complementary to the long-established role of progesterone in promoting post-ovulatory tissue remodelling in women. We demonstrate that androgens regulate *early* remodelling events by affecting endometrial transcription and altering the expression of decidualization and receptivity markers. These data suggest intra-uterine androgens may be critical for decidualization and thus impact on establishment and maintenance of pregnancy.

## Materials and Methods

### Patients, endometrial tissue resource and cell cultures

Human endometrial tissue (proliferative phase, n = 20) was obtained from women undergoing surgery for non-malignant gynaecological conditions. None of the women were receiving hormonal therapy or suffering from endometriosis. Cycle phase was determined as previously reported^37^. Written informed consent was obtained from all subjects prior to surgery, and ethical approval was granted by the Lothian Research Ethics Committee (LREC 10/S1402/59). Methods were carried out in accordance with NHS Lothian Tissue Governance guidelines. Primary endometrial stromal cells (ESC) were isolated from proliferative phase endometrium as described previously^37^. Decidualization was induced by addition of decidualization media (DM: RPMI 1640, 2% charcoal-stripped FCS, 0.1 mg/ml 8-Br-cAMP (Sigma B5386), 1 μM progesterone (Tocris, Cat no. 2835). Some cells were incubated with the antiandrogen flutamide for the duration of the culture period (10 μM; Sigma F9397). Control cultures were incubated with RPMI 1640, 2% charcoal-stripped FCS and equivalent volume of vehicle control (DMSO). To assess the time-dependent accumulation of secreted products treatments were maintained for the duration of each time point. ESC were treated for 1, 2, 4 and 8 days as indicated.

### Measurement of mRNA and protein

Isolation of mRNAs, preparation of cDNAs, and analysis by qRTPCR was performed according to standard protocols (11); samples were quantified by standard curve method or by the comparative ΔΔCt method with *CYC* as internal control. Primers/probes are given in Supplemental Table 1.

Western blotting was performed using 50 μg/lane total cell lysates; membranes were probed with rabbit or goat anti-SRD5A1 (Santa-Cruz Biotech; sc-20658 and sc-20396); rabbit anti-AKR1C3 (Abcam, Ab137546); rabbit anti-Monoamine Oxidase A (Abcam, ab126751) or rabbit anti-Endothelin B Receptor (Abcam ab129102). SRD5A1 was not detected at the predicted molecular weight (29 kDa) but consistently detected at around 100 kDa at all reducing conditions tested (50–250 mM dithiothreitol; DTT, β-mercaptoethanol and tributylphosphine; TBP). Identity of the protein was confirmed using two different specific antibodies; no bands were detected if the primary antibody was incubated with specific blocking peptide (SRD5A1 (A-18) peptide sc-20396 P) or in controls lacking the primary antibodies. In all cases loading control was goat anti-actin (Santa-Cruz biotech sc-1616; predicted molecular weight 43 kDa). Membranes were incubated with species-specific fluorescent-conjugated secondary antibodies and visualised using the Licor Odyssey system (Licor). Protein bands detected by Western blot were quantified by performing densitometry analysis using ImageJ (NIH.gov). Data were normalised to Actin and expressed as fold-change relative to control treatment.

### Immunofluorescence

Immunofluorescence was carried out on ESC grown on chamber slides (BD Biosciences) incubated with control media or DM to identify the localisation of the proteins within the cell and as numbers of primary cells were limited only one time point was selected (4 days). In line with expectations, both AKR1C3 and SRD5A1 were localised to the cytoplasm of the primary ESC a finding consistent with expression within the endoplasmic reticulum. Primary antibodies; rabbit anti-SRD5A1 (Santa-Cruz Biotech; sc-20658) and rabbit anti-AKR1C3 (Abcam, Ab137546) were incubated overnight at 4 °C. Antigen detection was performed using a Tyramide signal amplification (Perkin Elmer) system followed by SYTOXGreen nuclear counterstain (Life technologies). Images were captured using a LSM 710 Confocal microscope (Zeiss). Cell morphology was assessed by phase-contract imaging using Axiovert 200 inverted microscope (Zeiss) with 5× objective.

### ELISA

Testosterone (T), dihydrotestosterone (DHT) and Insulin-like Growth Factor Binding Protein 1 (IGFBP1) were measured in culture supernatants. Media were assayed in duplicate. Assay specificities are described in Supplemental Tables 2–4.

### Statistical analysis

Statistical analysis was performed using Graphpad prism. Student’s t test or One-way ANOVA were used to determine significance between treatments in data that were normally distributed. Non-parametric testing was utilised where sample sizes were insufficient to confirm normality of data distribution; Mann-Whitney test or Kruskal-Wallis test was used to assess differences between treatments. Where data were analysed as fold change significance was tested using one sample t test and a theoretical mean of 1. Criterion for significance was p < 0.05. All data are presented as mean ± SEM.

## Additional Information

**How to cite this article**: Gibson, D. A. *et al*. Intracrine Androgens Enhance Decidualization and Modulate Expression of Human Endometrial Receptivity Genes. *Sci. Rep*. **6**, 19970; doi: 10.1038/srep19970 (2016).

## Supplementary Material

Supplementary Information
