# Supplementary material for: Intracrine Androgens Enhance Decidualization and Modulate Expression of Human Endometrial Receptivity Genes
Source: Sci Rep. 2016 Jan 28;6:19970. doi: 10.1038/srep19970 (PMC4730211; doi:10.1038/srep19970)
Supplement: Supplementary Information [file srep19970-s1.pdf]

# Intracrine Androgens Enhance Decidualization and Modulate Expression of Human Endometrial Receptivity Genes.

Authors: Douglas A Gibson[1], Ioannis Simitsidellis[1], Fiona L Cousins[1*], Hilary O.D. Critchley[2] and Philippa T.K. Saunders[1]

Medical Research Council Centres for Inflammation Research[1] and Reproductive Health[2], The University of Edinburgh, Queen's Medical Research Institute, 47 Little France Crescent, Edinburgh, EH16 4TJ. UK

[*] Current address: The Ritchie Centre, Hudson Institute of Medical Research, 27-31 Wright Street, Clayton, Victoria, 3168, Australia.

**Supplementary Data:** 4 Figures and 4 Tables

**Supplementary Figure 1. Regulation of androgen metabolism in the endometrium.**

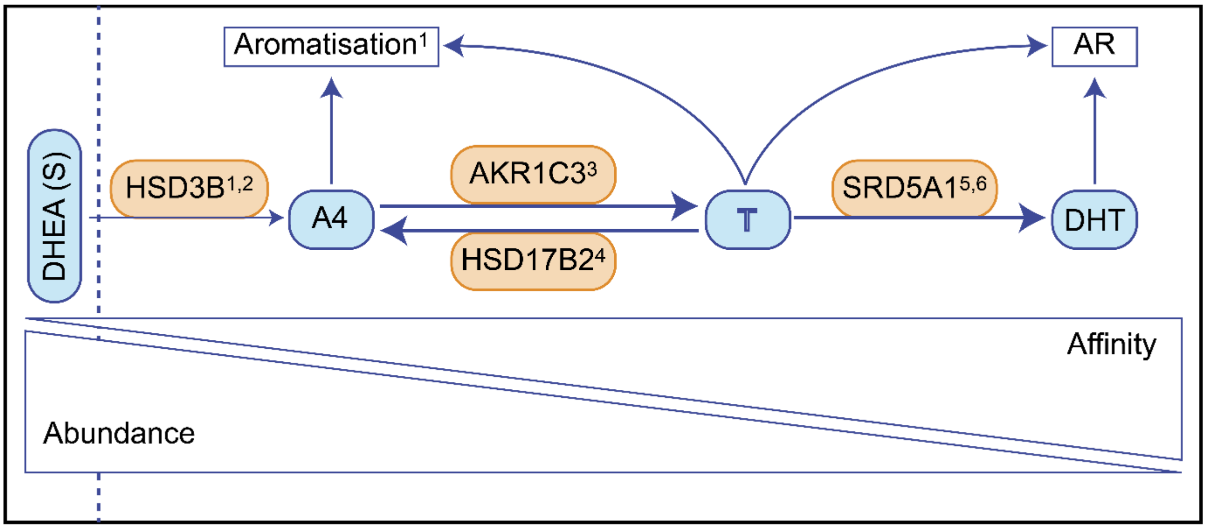


The enzymes required for the formation of bioactive androgens are reported to be expressed in the human endometrium. The adrenal androgen DHEA and its sulphate (DHEAS) are abundant in the circulation, DHEA can be converted to A4 by the enzyme HSD3B reported to be expressed in human ESC and in human endometrial tissue [1, 2]. A4 can be aromatised to estrogens [1] or converted to T by AKR1C3 which is reported to be expressed in human endometrium across the cycle with peak expression in the early secretory phase [3]. T can also be metabolised to A4 by the action of HSD17B2 [4] or converted to DHT by SRD5A1 [5, 6].


1. Gibson, D.A., et al., *Endometrial Intracrinology--Generation of an Estrogen-dominated Microenvironment in the Secretory Phase of Women.* The Journal of clinical endocrinology and metabolism, 2013. **98**(11): p. E1802-6.
2. Rhee, H.S., et al., *Expression of 3beta-hydroxysteroid dehydrogenase and P450 side chain cleavage enzyme in the human uterine endometrium.* Exp Mol Med, 2003. **35**(3): p. 160-6.
3. Catalano, R.D., et al., *Comprehensive expression analysis of prostanoid enzymes and receptors in the human endometrium across the menstrual cycle.* Mol Hum Reprod, 2011. **17**(3): p. 182-92.
4. Aghajanova, L., et al., *Steroidogenic enzyme and key decidualization marker dysregulation in endometrial stromal cells from women with versus without endometriosis.* Biol Reprod, 2009. **80**(1): p. 105-14.
5. Ito, K., et al., *Expression of androgen receptor and 5alpha-reductases in the human normal endometrium and its disorders.* Int J Cancer, 2002. **99**(5): p. 652-7.
6. Carneiro, M.M., et al., *Androgen receptor and 5alpha-reductase are expressed in pelvic endometriosis.* BJOG, 2008. **115**(1): p. 113-7.

**Supplementary Figure 2. Metabolism of testosterone**

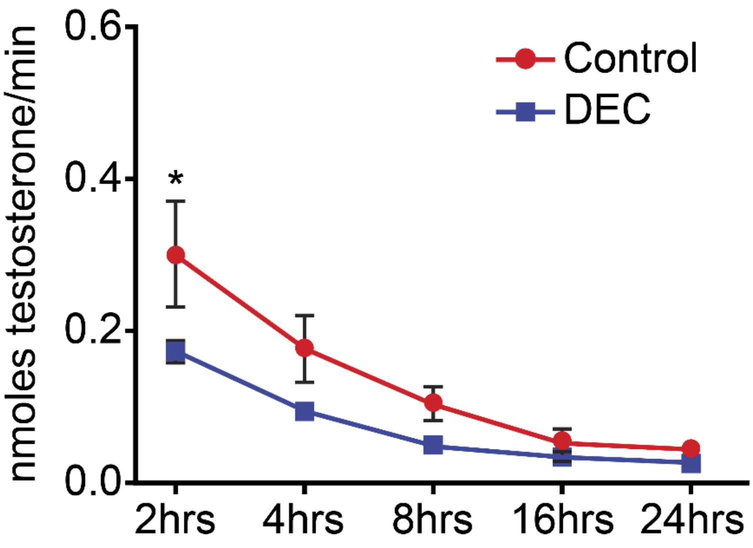


Metabolism of T was analysed by thin layer chromatography (TLC) as described previously [2]; cells were cultured with medium containing unlabelled steroid (T; 10-8M) and tritium labelled T for 24 hours. The rate of metabolism was determined as the percentage conversion of substrate (nmoles) over time in minutes. Metabolism of tritiated T to A4 was measured over 24 hours and was significantly decreased in decidualized (DEC) compared to control ESC (Ctrl, n=4, $p<0.05$).

**Supplementary Figure 3. The impact of flutamide on secretion of putative androgen-regulated receptivity marker SPP1.**

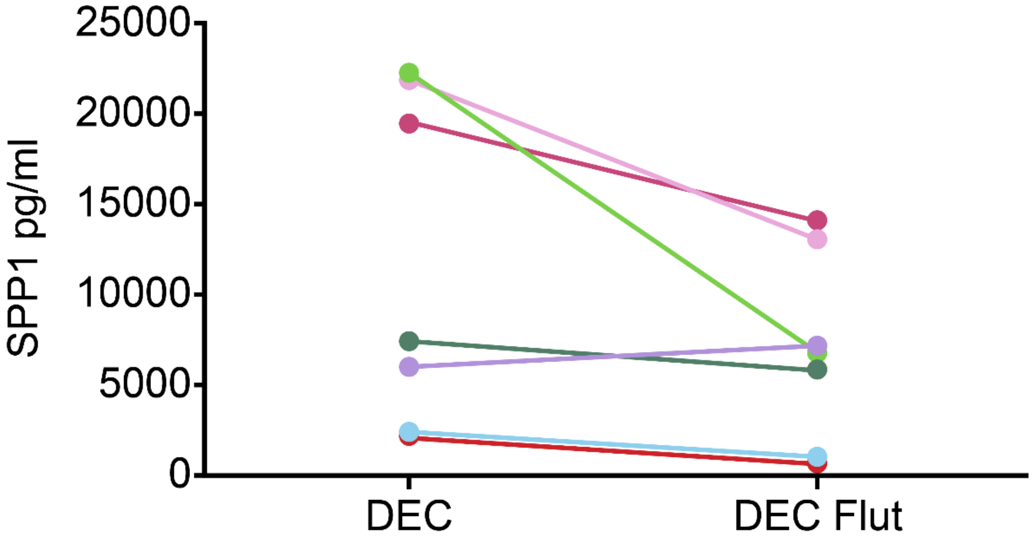


The concentrations of SPP1 in cell culture supernatants from ESC treated to decidualize (DEC) and those co-treated with the antiandrogen flutamide (DEC Flut) was assessed by ELISA. Flutamide treatment tended to reduce of SPP1 but a broad range of concentrations were detected between in individual patients (n=8 patients). Matched values for individual patients are shown identified by different colours for each paired patient measurement.

**<u>Supplementary Figure 4. Expression of candidate androgen-regulated receptivity factors after 4 days treatment determined by Western Blot.</u>**

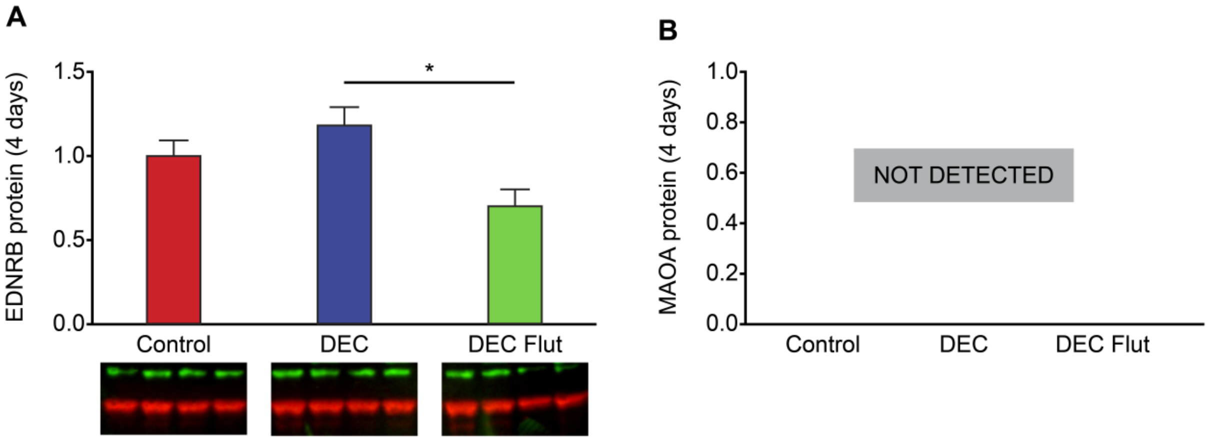


**A** Western blot analysis of EDNRB expression from homogenates of ESC treated for 4 days revealed that EDNRB protein in was detected in control and decidualized ESC (n=8 patients per treatment), and tended to be increased in ESC treated to decidualize. Co-treatment with flutamide significantly reduced concentrations EDNRB detected relative to decidualized ESC (n=8, * p<0.05). Representative blots from 4 matched patients are shown. Loading control b-actin (red, 43kDa), EDNRB (green, 50 kDa). **B** MAOA was not detected in ESC homogenates treated for 4 days.

**Supplementary table 1 -** Primers and probes for qRTPCR

| Gene Symbol | 5' to 3' | 3' to 5' |
|---|---|---|
| *AKR1C3* | tgggttccgccatatagatt | tcgatgaaaagtggaccaaa |
| *EDNRB* | atcgtcattgacatccctatca | gcttacacatctcagctccaaa |
| *IGFBP1* | aatggattttatcacagcagacag | aatggattttatcacagcagacag |
| *MAOA* | ggccacatgttcgacgtagt | catgccaatctcttctcttgg |
| *PRL* | aaaggatcgccatggaaag | gcacaggagcaggtttgac |
| *SPP1* | gagggcttggttgtcagc | caattctcatggtagtgagttttcc |
| *SRD5A1* | catgttcctcgtccactacg | aacataatcgccattgtacacg |
| *TF3* | ggagaaaggggaattcagaga | gggagttctccttccagctc |

**Supplementary Table 2** – Assay performance

| ELISA | Intra-assay CV (%) | Inter-assay CV (%) | Sensitivity |
|---|---|---|---|
| IGFBP1 | 2.4-10.2 | 5.5-8.7 | 31.2 pg/ml |
| Testosterone | 6.6-9.6 | 6.1-8.5 | 0.022 ng/ml |
| Dihydrotestosterone | 3.9-11.4 | 5.9-12.1 | 6.0 pg/ml |

**Supplementary Table 3** - Cross-reactivity of testosterone ELISA

| Steroid | % Cross-Reactivity |
|---|---|
| Testosterone | 100 |
| 5α-DHT | 5.2 |
| Androstenedione | 1.4 |
| Androstanediol | 0.8 |
| Progesterone | 0.5 |
| Androsterone | 0.1 |

**Supplementary Table 4** – Cross-reactivity of dihydrotestosterone ELISA

| Steroid | % Cross-Reactivity |
|---|---|
| Dihydrotestosterone | 100 |
| Testosterone | 8.7 |
| 5β-dihydrotestosterone | 2.0 |
| Androstenedione | 0.2 |
